# The Docosahexanoic Acid: From the Maternal-Fetal Dyad to Early Life Toward Metabolomics

**DOI:** 10.3389/fped.2020.00538

**Published:** 2020-09-30

**Authors:** Federica Comitini, Chiara Peila, Vassilios Fanos, Alessandra Coscia

**Affiliations:** ^1^Neonatal Intensive Care Unit, Department of Surgical Sciences, University Hospital and University of Cagliari, Monserrato, Italy; ^2^Complex Structure Neonatology Unit, Department of Public Health and Paediatric, University of Turin, Turin, Italy

**Keywords:** docosahexanoic acid (DHA), human milk, metabolomics, infant nutrition, preterm nutrition

## Abstract

Docosahexaenoic acid (DHA) is an essential ω-3 long-chain polyunsaturated fatty acid (LCPUFA) and represents the dominant structural fatty acid in the retina and in the brain's gray matter. Due to its active participation in the development of the nervous system, DHA is one of the most studied LCPUFA and is currently considered a critical nutrient during pregnancy and breastfeeding. Increasing evidence in literature suggests that an adequate concentration of DHA is required from the fetal stage through to early life to ensure optimal neurological development. Likewise, many studies in literature demonstrated that an adequate supply of DHA during pregnancy and lactation is essential to promote proper brain development in utero and in early life. Daily supplementation of DHA in newborns has potentially stronger effects compared to maternal supplementation during pregnancy. Supplementation initiated in the second year of life in children born preterm did not result in global cognitive development improvements. Preliminary findings arising from metabolomics has reported that mother's milk and infant formula supplementation of Vitamin D associated with DHA results in a higher antioxidant and protective action, with a possible positive influence on renal function and body fat on preterm infants compared to those receiving only vitamin D. Recent applications of metabolomic studies on newborns may lead to a better understanding of the metabolic process linked to early nutrition and, subsequently, to the development of targeted and personalized nutritional strategies.

## Introduction

Docosahexaenoic acid (DHA, C22:6*n*−3) is an essential ω-3 long-chain polyunsaturated fatty acid (LCPUFA) and represents the dominant structural fatty acid in the retina and in the brain's gray matter (1). DHA accounts for 50% of the total brain lipids and between 60 and 80% of the brain membrane phospholipids (2) and is the most abundant *n*−3 LCPUFA in the nervous system (NS). In literature, its function has been shown as crucial for neurogenesis and synaptogenesis, taking an important role in neurotransmitter function, signal transduction, gene expression, neurogenesis, and anti-inflammation (3). Due to its involvement in the development of NS, DHA is one of the most studied LCPUFA and is currently considered a critical nutrient during pregnancy and breastfeeding (4).

### Pregnancy

The development of the NS starts in utero and proceeds until the postnatal period. During the last trimester of pregnancy, the so-called “brain growth spurt” takes place, i.e., a period in which the brain's growth is very fast, and its composition quickly changes (5). DHA is integrated into brain and retinal cell membranes in the third trimester of pregnancy and the first two years of life (6). During pregnancy, LCPUFAs needs for the fetus are elevated and mainly satisfied by the placenta, with only a little amount synthesized into the fetus (7). For this reason, the concentration of fetal DHA is correlated to the amount of DHA ingested by the mother (8). Adequate nutrition during pregnancy is thus essential, and maternal DHA intake during pregnancy is associated with the child's neuro-developmental performance (9, 10). Reports in literature have shown how women from Western countries consume diets that are low in DHA, which is almost exclusively present in seafood and accumulates throughout the marine food chain from sources such as microalgae (11). Moreover, pregnant women could reduce their consumption of certain species of deep-sea predatory fish due to the potential presence of heavy metals like mercury, lead, and cadmium (3). Literature data suggest that only a small quantity of DHA can be synthesized in the body from α-linolenic acid (ALA), a shorter chain omega-3 fatty acid that can be found in many vegetables and plants commonly eaten (12).

Docosahexaenoic acid supplementation in the prenatal period has also been associated with a decreased incidence of allergic diseases in infants (13). A report published in 2013 by Kuratko et al. (14) demonstrated that a supplement of 600 mg DHA/d in the last half of gestation resulted in overall greater gestation duration, with reduction in early preterm and very-low birth weight (VLBW) births (14). One mechanism by which DHA may decrease the incidence of preterm birth is by decreasing prostaglandin E2 and prostaglandin F2α production, thereby reducing inflammation within the uterus, which is a risk factor for preterm birth (15).

In a recent article published by Feltham et al. (16) DHA supplementation in pregnant women was reported to have a potential role in combating prenatal alcohol-induced insults on brain development.

The above findings suggest that it is necessary to promote supplementation with DHA during pregnancy and lactation through consuming products rich in DHA, such as fish oil, krill oil capsules, or food fortified with *n*−3 LCPUFA (17). While in the intra-uterine life the availability of DHA for the infant is directly related to transfer through the placenta, after birth it depends on transfer through lactation (18).

### Lactation

Human milk is a common and natural source of LCPUFA and DHA.

PUFA concentration in human milk is relatively stable. DHA concentration shows a decrease (approximately from 0.5% colostrum to 0.25% mature milk) according to the stage of lactation (19–21). DHA levels in HM are also influenced by gestational pathologies. Three studies evaluated the fatty acid composition of the HM of Preeclamptic mothers (PE). Fares et al. (22) evaluated the total fatty acid profile of colostrum in mothers who delivered preterm and found lower DHA levels and higher Arachidonic Acid (AA) levels, AA:DHA, and ω6:ω3 ratios in the PE group. Dangat et al. (23, 24) published two studies focused on long chain polyunsaturated fatty acids. The first study shows that DHA and nervonic acid concentrations were higher in the colostrum of the PE group, as compared to the normotensive control group. The second study shows that DHA concentrations were also higher at day 3, and at 1.5 and 3.5 months (23).

The DHA level of human milk varies widely across different countries and regions; a possible factor contributing to this difference is the diet. The DHA levels range between 0.17 and 0.99% of total fatty acids, with the lowest levels reported for both Canada and the United States and the highest levels in Japan. High levels (0.74% of total fatty acids) were also reported for the Philippines. A 2007 review by Brenna et al. reported that “a mean DHA human milk level of 0.32% (25). For DHA, the lowest value (0.06%) was reported for Pakistan, but similar low values were observed in rural South Africa (0.1%) and Canada (0.12%), whereas the highest values were reported for the Canadian Arctic (1.4%) and Japan (1.1%).”

The latest research from Fu et al. (26) and Sánchez-Hernández et al. (27) assessed that: “the DHA and arachidonic acid (AA) levels in human milk (vary) according to country and region, reporting similar values (0.42% for DHA and 0.71% for AA).”

Literature data often report that *n*−3 fatty acids such as DHA are modulated by diet whereas *n*6 fatty acids, like ARA, are not (28).

Breastfeeding women need to consume a daily DHA intake of 200 mg to produce milk with a DHA content of at least 0.3%. This DHA concentration is required for a breastfed infant to obtain the daily DHA supply considered desirable to meet metabolic needs (100 mg DHA/day) (29). In recent years, milk companies have started to enrich infant formulas with DHA, but literature data suggest that DHA supplementation may be associated with either better outcomes or with no effect in infants fed artificially. This probably depends on DHA levels in formulas, on the co-presence of Arachidonic acid (ARA), on infant DHA status at birth, and on the infant's genetic background (9). On the other hand, neonates fed with enriched formula have a lower incidence of infections and allergic/atopic diseases in early stages of life, compared to exclusively breast-fed infants (30).

### Neonatal Period

Preterm infants, especially those born very preterm, do not have the opportunity to accumulate DHA during most of the last trimester of pregnancy due to a shortened gestation (31).

During neonatal life, preterm infants require more nutrients to ensure the maturation and development of tissues and organs. LCPUFA needs are also higher (7). Furthermore, enzymes responsible for the endogen synthesis of DHA in preterm newborns have low activity (32). Consequently, preterm infants are disadvantaged compared to term infants for access to the DHA needed for brain maturation. This factor exposes these infants to a higher risk of neonatal morbidities (7). Several studies demonstrated that DHA daily supplementation begun in the neonatal period improves global developmental outcomes in preterm infants. Moreover, recent studies show that higher circulating DHA levels in premature infants are related to better vision and neurodevelopmental outcomes (33). High DHA levels also correlate with a lower risk of necrotizing enterocolitis (34), retinopathy of prematurity (35), and bronchopulmonary dysplasia.

The metabolomics approach has been recently applied in a study investigating the dynamic variations in the urinary metabolic profiles of two groups of preterm infants receiving different supplementation strategies (36). Supplementation was focused on increasing DHA and vitamin D content in mother's milk or infant formula over a period of two months after hospital discharge. The first group of preterm newborns received only 400 I.U./day of vitamin D (DS), whereas the second group received a supplementation of vitamin D enriched with 20 mg/day of DHA (D-DHAS). In this study, significant differences between the two types of supplementation came to light. Interestingly, compared to the DS group, the D-DHAS group exhibited more abundant urine levels of betaine, creatinine, N, NDMG, and DMA, and a lower content of myo-inositol and lactate (36). Researchers found that lower values of myo-inositol and lactate in urine are related to lower cellular and organism stress, especially for the brain (37), while N,N-DMG protects the neurodevelopment and the neuronal cell function thanks to its antioxidant function (37). In an animal study on DMA, a product of choline metabolism by the gut microbiota is reported to be inversely correlated with body fat (38). The increasing rate of urinary excretion of creatinine, which in previous studies has been positively correlated with weight, height, and postconceptional age, can also be associated with the maturation of infants' renal functions (39). Betaine is synthesized by choline dehydrogenase and betaine aldehyde dehydrogenase in the gut mucosa, liver, and kidney (40). It is widely recognized as an osmolyte or methyl group donator to homocysteine to form methionine and S-adenosylmethionine, promoting remethylation (41).

Overall, the results of this study suggest higher antioxidant and protective action by supplementation with DHA and vitamin D compared to those with only vitamin D and a possible positive influence on renal function and body fat composition (41).

The current standard of supplementation in preterm newborns is enteral feeding and the standard dietary provision of DHA is based on the average worldwide content found in human milk (4), which is 0.3% of total milk fatty acid (4, 42). Regardless, formula or breast milk DHA supplementation remains dependent on the infant's ability to tolerate full volume enteral feedings, and the optimal dose and method of administration is still unknown (42). A randomized clinical trial published in 2018 by Keim et al. (43) reported that DHA daily supplementation did not result in improvements in cognitive development in preterm newborns if started only in the second year of life. The meta-analysis conducted in 2018 by Shulkin reviewed the randomized controlled trials of *n*−3 PUFA supplementation in mothers or infants (age ≤ 2 years) and evaluated standardized measures of cognitive or visual development up to 18 years of age. The authors found that *n*−3 PUFA supplementation improves visual acuity and MDI in preterm infants, without statistically significant effects on PDI or IQ in different intervention period subgroups (6).

## Discussion and Conclusion

Increasing evidence in the literature suggests that an adequate concentration of DHA is required from the fetal stage to early life to ensure optimal neurological development.

Docosahexaenoic acid supplementation is reported to have benefits on pregnancy outcomes; therefore, pregnant women should be supported to achieve a DHA intake of 600 mg daily in the last trimester of pregnancy.

The third trimester experiences the highest DHA accumulation, and premature infants are therefore at risk of DHA deficiency due to a shortened gestation. For this reason, they should receive a diet with at least 0.3% DHA (4), but further trials are necessary to determine the optimal dose and route of administration of DHA in newborns.

Daily supplementation of DHA in newborns has potentially stronger effects compared to maternal supplementation during pregnancy. In children born preterm, supplementation initiated only in the 2nd year of life did not result in a global cognitive development improvement.

Preliminary findings arising from metabolomics suggest that mother's milk and infant formula supplementation of Vitamin D associated with 400 I.U./day of DHA result in a higher antioxidant and protective action compared to those receiving only vitamin D. A potential positive influence on renal function and body fat on preterm infants was also found. Recent applications of metabolomic studies on newborns may lead to a better understanding of the metabolic processes linked to early nutrition and, subsequently, to developing targeted and personalized nutritional strategies.

## Author Contributions

FC and CP have made substantial contributions to conception and design and has been involved in drafting the manuscript. VF anc AC have been involved in drafting the manuscript and revising it critically for important intellectual content. All the authors read and approved the final version of the paper.

## Conflict of Interest

The authors declare that the research was conducted in the absence of any commercial or financial relationships that could be construed as a potential conflict of interest.
